# Biosafety Level 3 setup for multiphoton microscopy *in vivo*

**DOI:** 10.1038/s41598-017-00702-x

**Published:** 2017-04-03

**Authors:** D. Barlerin, G. Bessière, J. Domingues, M. Schuette, C. Feuillet, A. Peixoto

**Affiliations:** 1 Centre National de la Recherche Scientifique (CNRS), Institut de Pharmacologie et de Biologie Structurale (IPBS), 31000 Toulouse, France; 2 Université de Toulouse, Université Paul Sabatier, Institut de Pharmacologie et de Biologie Structurale (IPBS), 31000 Toulouse, France; 3 EuroBioconcept SAS, 94380 Bonneuil sur Marne, France; 4 Lavision Biotech GmbH, 33617 Bielefeld, Germany

## Abstract

Multiphoton microscopy has revealed important insights into cellular behavior *in vivo*. However, its application in infectious settings often encounters technical, safety and regulatory limitations that prevent its wider use with highly virulent human pathogens. Herein, we present a method that renders multiphoton microscopy *in vivo* compatible with biosafety level 3 regulations and present an example of its application and potential to visualize a *Mycobacterium tuberculosis* infection of the mouse lung.

## Introduction

Multiphoton microscopy (MPM) has revealed important insights into cell-cell interactions and dynamic behavior of cells *in vivo*, which is due to its ability to provide four-dimensional (x, y, z and time) imaging of tissues over depths of several hundreds of microns while limiting phototoxicity and photobleaching^1^. For example, MPM has revealed an unsuspected fast and random migratory behavior of lymphocytes in lymph nodes and opened several new lines of research^2–4^. The use of MPM in non-infectious settings poses a minimal biological safety risk to the user or the environment. However, its application in infectious settings exposes both to a risk that is related to the virulence of the microorganism at study. Microorganisms are classified according to their virulence, which then establishes the biosafety level (BSL) of containment necessary for the work with each microorganism. These different levels of containment are BSL1 for agents not associated with disease in healthy individuals; BSL2 for agents associated with disease, but rarely serious, for which treatments/vaccines are available; BSL3 for agents associated with serious/lethal disease for which treatments/vaccines are available and BSL4 for agents associated with serious/lethal disease for which treatments/vaccines are not usually available^5^. To perform MPM studies with BSL3 agents, the laboratory is placed under negative pressure relative to the exterior, thus preventing any contamination from exiting the laboratory. Inside a BSL3 laboratory the contact of the user with the pathogen is prevented by Personal Protective Equipment (PPE), Standard Operating Procedures (SOP) and by physical barriers in order to minimize the risk of exposure. Moreover, since laser and microscope manufacturers frequently do not perform interventions within operating BSL3 laboratories, the MPM equipment needs to be devoid of any contamination inside the facility or remain fully functional after undergoing a decontamination procedure prior to being serviced. Currently, while there is a strong interest in studies of highly virulent human pathogens (e.g. *Mycobacterium tuberculosis*, a BSL3 agent) that are a significant threat to public health, MPM studies are extremely rare^6, 7^. Our belief is that studies applying MPM techniques in BSL3 settings remain rare due to the lack of standardized biosafety level 3 containment methodology to perform such studies. In contrast, several MPM *in vivo* studies with more tractable BSL1 and BSL2 microorganisms have been reported^8–14^.

In the field of tuberculosis *in vivo* probing of immune cell and pathogen behavior by MPM techniques in combination with mouse lines in which specific cell types are fluorescent (e.g. Macblue^15^, LysM-GFP^16^, CD11c-YFP^17^, CX3CR1-GFP^18^) holds great promise of providing key insights into the host/pathogen interactions that are behind the success of *M*. *tuberculosis* in persisting in humans. Indeed, several aspects in the animal models of infection and tuberculosis patients that are related to cell-cell interactions and cell migration and believed to contribute to *M*. *tuberculosis* virulence remain unclear. For instance, alveolar macrophages are thought to phagocytose *M*. *tuberculosis* upon entry in the airways but the transport of live bacilli from the lung to the draining lymph nodes is performed by monocytes, which leads to the activation of naïve T cells. Yet this transport is significantly delayed in comparison to other infections and leads to a late generation of effector T cells from naïve T cells that are critical for the control of infection^19–21^. A characteristic that can also be observed in tuberculosis patients^22, 23^. So far the mechanisms leading to the cell-cell spread of *M*. *tuberculosis* and the fact that alveolar macrophages or dendritic cells do not transport the bacilli to lymph nodes requires further investigation. Furthermore, while the arrival of effector T cells in the infected lung is associated with the control of bacterial replication it does not lead to the clearance of infection^24^. A prior study has shown by MPM that effector T cells arrest poorly in the proximity of *M*. *tuberculosis* infected cells in the liver and show very limited cytokine production, which may explain the poor control of infection since these are critical factors for efficient T cell effector function^25, 26^. However, it is not clear if a similar phenomenon can be observed in the lung and if the behavior of T cells depends on their status of T cell differentiation. Indeed, it is possible that memory T cell subsets (central, effector or tissue-resident memory) that are generated after vaccination^27^ may have different behaviors in the context of *M*. *tuberculosis* infection and these may be related to their different protective capacities against infection.

In order to unlock the use of MPM approaches in BSL3 settings, we have developed a novel method to adapt the instrumentation for multiphoton microscopy to respect biosafety regulations and help driving *in vivo* imaging studies of human pathogens. In addition, we show the potential of our method for probing immune cellular behavior during *M*. *tuberculosis* infection in the mouse lung.

## Results

### Biosafety Cabinets for multiphoton microscope and Ti-Sapphire laser components

The instrumentation necessary to perform MPM consists in a microscope equipped with optics to illuminate the specimen and guide reflected light into the detectors, which is coupled to a femtosecond pulsed laser source. MPM studies in a BSL3 setting are challenging due to the fact that in both microscope and lasers parts several components are sensitive to decontamination agents, e.g. vaporized hydrogen peroxide, that are used eliminate any microorganisms in the instrumentation before any repair or maintenance intervention. Moreover, laser manufactures do not perform servicing inside BLS3 laboratories. Hence, our strategy to adapt a MPM setup to a BSL3 setting consisted in developing a methodology to prevent the contact of sensitive parts with microorganisms and allowing the removal of laser components from laboratory in order to perform servicing in a non-infectious facility. For this we employed a two-pronged approach for biosafety confinement of the MPM setup. First, we built a custom-made Class III Biosafety Cabinet (BSC) under negative pressure to confine the infectious material being imaged and to prevent any contamination from reaching the other parts of the microscope. For this a compromise was made since transmitted light could not be used in our system. Second, we confined the laser components within a modified Class I BSC under positive pressure with HEPA filtered air, thus preventing any contamination to reach any of its parts, even in the case of a leak on the class III BSC or an incident within the room.

**Figure 1:**
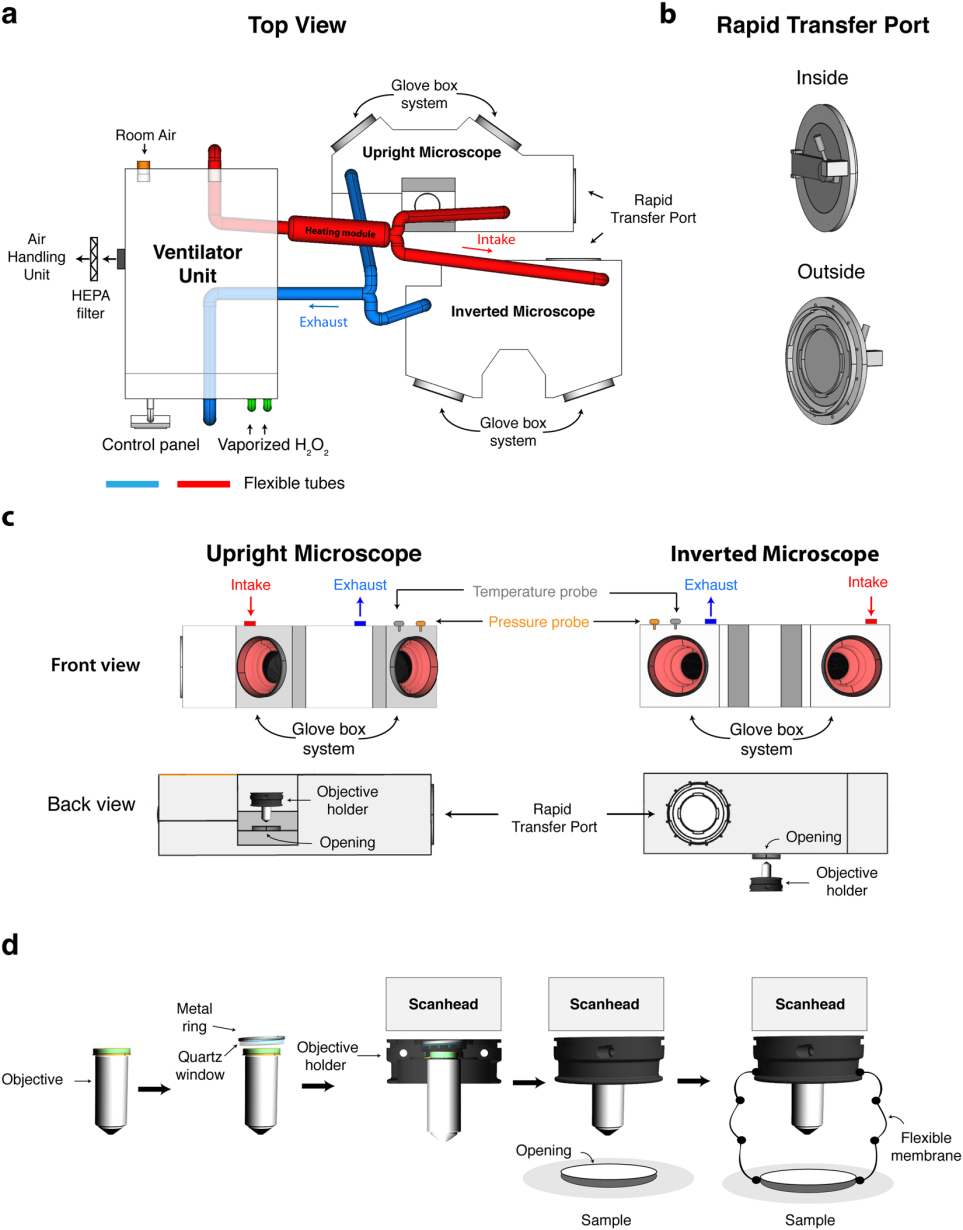
Class III Biosafety Cabinet for inverted and upright multiphoton microscope. (**a**) The ventilator unit extracts room air that is HEPA filtered and pulsed into to the heating module before reaching the isolators by means of PVC flexible tubes (red). A dedicated air-handling unit extracts the air within the BSCs through PVC flexible tubes (blue) that is HEPA filtered before joining the laboratory exhaust air for final HEPA filtration. For decontamination of the BSC, a H_2_O_2_ generator is connected to the ventilator unit by a camlock fitting (green) and the vaporized H_2_O_2_ follows the same circuit as described above. A digital control panel is used to setup pressure, temperature and the decontamination cycle. (**b**,**c**) Both inverted and upright BSC are outfitted with a Rapid Transfer Port, a glove box system, a temperature probe, a pressure probe as well as an opening for the objective. (**d**) Behind the objective an optical quartz window is placed and tightened into place using a threaded 42 mm metal ring that compresses a 38 mm O-ring. To ensure a seal of the BSC at the level of the objective we connected the objective holder to the BSC by a flexible PVC membrane.

To build a class III BSC, we used 8 mm thick sheets of impact resistant Unplasticised Polyvinyl Chloride (PVC-U) assembled together by a plastic welding technique (Fig. 1a, Supplementary Figs S1 and 2). This class III BSC is used to confine the infectious material during MPM imaging and is fixed to the top of the microscope stage by 3 self-sealing screws. In addition, we outfitted the BSC with a 160 mm Rapid Transfer Port (RTP) and a glove system that ensures the entry/exit and handling of infectious material within the class III BSC without disruption of containment (Fig. 1b,c). The shape of the class III BSC was designed to permit an overall movement of 50 mm in both *x* and *y* axis and 80 mm in the z axis for the microscope stage, without touching any fixed parts of the microscope (e.g. eyepiece and objective holder). To provide access of the objective inside the class III BSC without disruption of containment, we built a custom-made objective holder that also serves as an attachment point for a flexible PVC membrane, which connects both parts (Fig. 1d and Supplementary Fig. S3). The final seal of the Class III BSC is ensured by placing an optically transparent (200–2500 nm, AR coated 600–1050 nm) quartz glass optical window behind the objective and compressing against it a 38 mm rubber O-ring by fastening a 42 mm threaded metal (Fig. 1c). More information on the dimensions of all BSC components can be found in Supplementary Figs S2 and S3 using the 3D measurement tool. In order to maintain the physiology of the tissue being imaged within the apparatus, the BSC is connected to a ventilation unit equipped with 2 High-Efficiency Particulate Arrestance (HEPA) filters to ventilate temperature regulated (32–37 °C) air. To prevent any escape of infectious material in case of a breach in the BSC, an extraction unit (0–150 m^3^/h) is used to create a negative pressure within this BSC. In addition, the ventilation unit has 2 camlock fittings to circulate vaporized hydrogen peroxide (H_2_O_2_) produced by an external generator for decontamination of the interior of the class III BSC. In our hands, vaporization of 450 ppm of H_2_O_2_ for 2 hours efficiently eliminates 2.2 × 10^6^ colony forming units (cfu) of *Geobacillus stearothermophilus* spores (ATCC #12980), positioned at 5 different positions within this class III BSC. Hence, this BSC Class III can be efficiently decontaminated since G. *stearothermophilus* spores are one of the most heat-resistant of aerobic microorganisms and are frequently used in the field to assess the efficiency of decontamination procedures^28^. Finally, a touch screen control panel is used to setup pressure, temperature and decontamination cycles within the class III BSC. Of note, in order to facilitate servicing of the MPM setup, the class III BSC can be disconnected by removing the 3 self-sealing screws that fix it to the microscope stage, disconnecting the air intake and exhaust flexible tubes and temperature and pressure probes.

**Figure 2:**
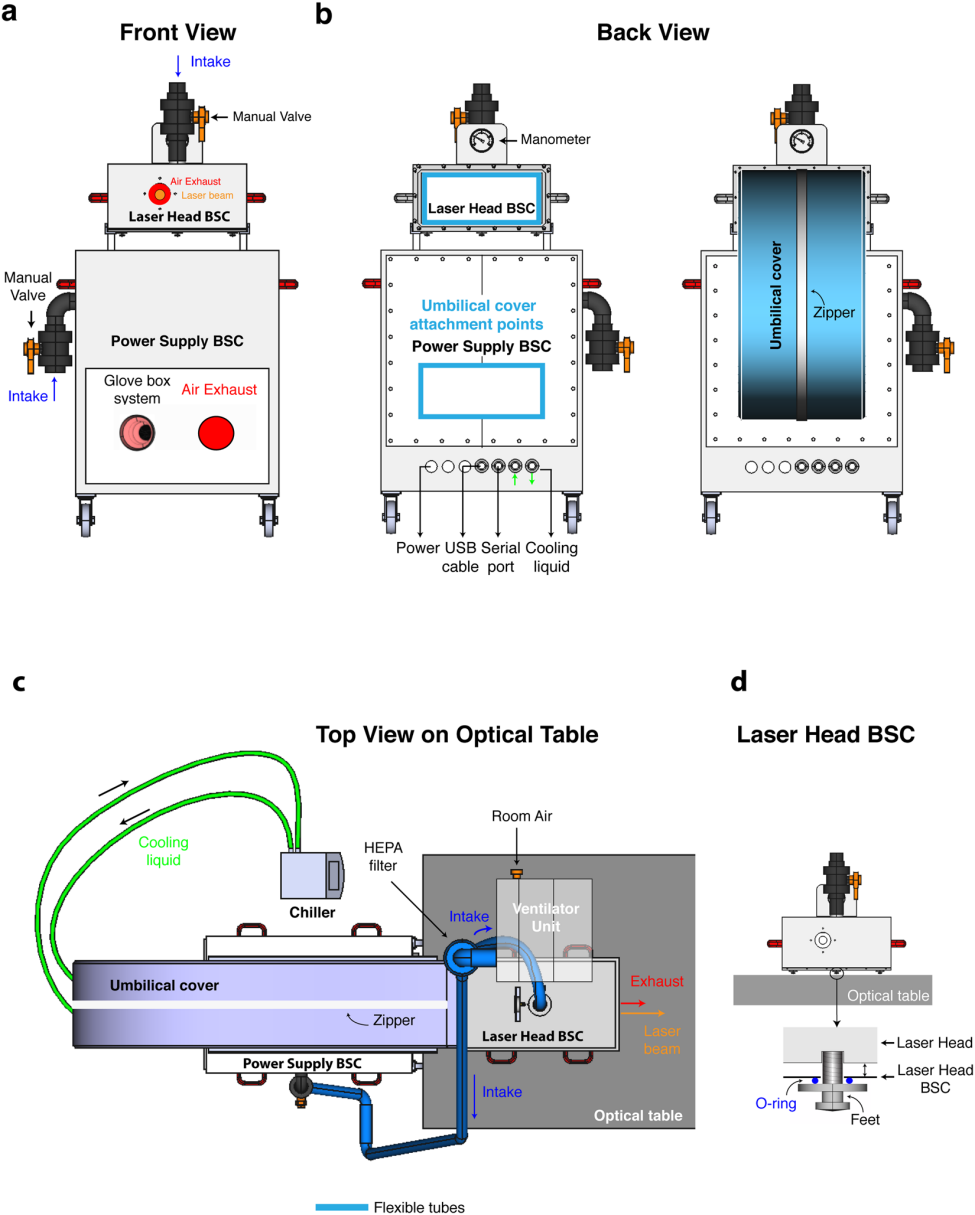
Class I Biosafety Cabinets for laser head and power supply of a Ti-Sapphire laser. (**a**) Front view. The laser head and power supply BSC are outfitted with a manual valve for the intake of air from a ventilator unit that maintains a positive pressure within each of them. The laser head BSC has built-in an opening that allows air exhaust and the exit of the laser beam. The power supply BSC has built in a glove box system to allow the access to the controls and an air exhaust. For convenience the laser head can rest on a rail of the power supply BSC for moving both parts within the BLS3 laboratory. (**b**) Back view. The power cables of the laser components are connected to a power bar and its cable pass through the wall of the power supply BSC by a thermos-retractable airtight cable gland before being plugged into a wall socket. The same type of pass-through connection is used to exit a USB and serial port cable from the power supply BSC to connect to a spectrometer and the computer piloting the microscope, respectively. A push-to-connect fitting housed within an airtight thermo-retractable cable gland does the connection of the laser head to the chiller by passing through the wall of the power supply BSC. (**c**,**d**) The laser head BSC sits on top of an optical table through the use of 3 custom made self-sealing feet that transverse the BSC to prevent its deformation overtime due to the weight of the laser head.

To prevent the laser from being exposed to contamination within the BSL3 laboratory, we built a modified Class I BSCs from 10 mm PVC-U using a plastic welding technique for both the laser head and the power supply. Both of these BSCs are under positive pressure created by an influx of HEPA-filtered air originating from a common ventilator unit (Fig. 2a, Supplementary Figs S1 and 2). The level of positive pressure (typically 50–100 Pa) within the BSC is ensured by two motors (1 main and 1 backup) inside the ventilator unit that can be monitored by a manometer placed in the laser head BSC. For convenience, the laser head BSC can be placed on a rail installed at the top of the power supply BSC, allowing their movement as unit within the BSL3 laboratory. The laser head BSC was built with an opening in the front panel for exhaust air and exit for the laser beam, as well as a manual control valve in the top panel to connect/disconnect it to/from the ventilator unit. The BSC for the power supply is equipped with a manual control valve in the side panel to connect/disconnect it to/from the ventilator unit, and a glove system to allow access to the controls of the power supply and an air exhaust in the front panel. The use of two air exhausts and high airflow rate is critical to maintain the temperature of the laser components according to the manufacturer’s specifications. Both BSCs have an opening in the back panel to allow the passage of the cables connecting the laser head to the power supply, chiller and miniature recirculator unit (MRU) (Fig. 2b, Supplementary Figs S1 and 2). While maintaining a proper seal, the power supply BSC also has built-in several airtight thermo-retractable cable gland connections in order to link: i/the MRU unit and power supply to a wall power socket; ii/the power supply to the computer piloting the MPM setup by a serial port; and iii/the laser head to a spectrometer by a USB port. Finally, the recirculation of the cooling fluid from the chiller within the laser head is made possible by a push-to-connect fitting housed within an airtight thermo-retractable cable gland. Since the umbilical cord connecting the laser head and the power supply cannot be disconnected, we used a flexible PVC membrane fitted with an impermeable zipper to enclose all cables and attached it to the back panel of each BSCs by multiple layers of stretch resistant and impermeable tape. This umbilical cover is important to allow free and independent movement of both BSCs while preventing any contamination to reach the laser components when positioning the laser on the optical table (Fig. 2c). Importantly, to prevent deformation of the PVC material over time and provoke misalignment of the laser beam with the microscope, the laser head rests directly on the optical table by 3 custom-made self-sealing feet that transverse the BSC (Fig. 2d). Overall, this setup is not only important for preventing the contamination of the laser components, but also to protect it from the decontamination procedure when entering/exiting the BSL3 laboratory through an H_2_O_2_ Safety Access System (SAS). Given that the consequences of a direct exposure of the laser components to vaporized H_2_O_2_ (or other sterilizing agents) remain unknown but mostly likely deleterious, our setup was built to allow the complete seal of both BSC units by closing the exhaust ports with self-sealing plates, and closing the intake ports from the ventilator unit and Chiller.

### Experimental validation of BSL3 setup for multiphoton microscopy

**Figure 3:**
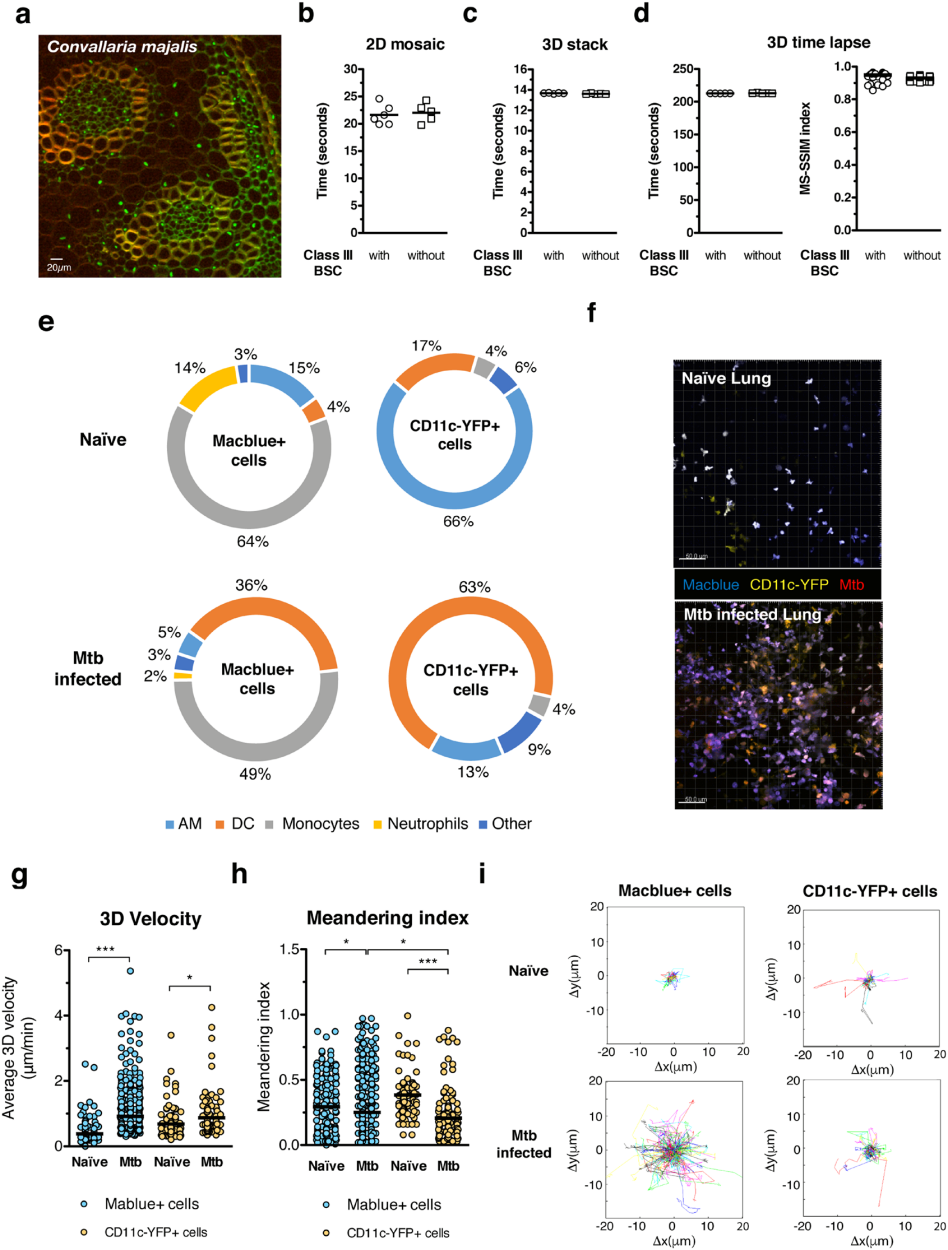
BSL3 setup for multiphoton microscopy: performance and applications. (**a**) A *Convallaria majalis* rhizome slide was used to evaluate the performance of the multiphoton microscope with and without the class III BSC installed. For this images with a size of 505*505 pixels were acquired with 3% of Ti-Saphire laser power tuned to 800 nm and with a pixel dwell time of 2,78 μs. These images were acquired either in 9 different x, y axis positions using the mosaic function (**b**), 13 different z axis positions with a step of 2 μm using the 3D stack function (**c**) and 13 z axis positions repeated overtime 15 times (**d**). Performance of x, y and z axis movement of the microscope was evaluated by recording the time necessary for completion of the different tasks. To quantify image stability overtime we calculated the Structural SIMilarity (SSIM) index between the first 3D stack and each of the subsequent ones within the same time-lapse sequence. Acquisition speed and stability values were calculated from 5 different acquisitions. (**e**) Flow cytometry analysis of naïve and *M*. *tuberculosis* (Mtb) (H37Rv) mouse lung according to the expression of Macblue and CD11c-YFP transgene. (**f**) Maximum intensity projection of a 3D stack from a naïve and Mtb infected lung vibratome slice. 3D velocity (**g**), meandering index (**h**) and Cell Tracks (**i**) of Macblue+ and CD11c-YFP+ lung cells in vibratome slices. Cell motility parameters were calculated in movies from 3 different areas of lung. Un paired t test was used to evaluate significant differences. *p < 0,05, **p < 0,01, ***p < 0,001.

Our BSL3 setup to perform MPM described above may impose constraints onto the imaging system that could negatively affect its performance. Indeed, supplemental weight of the class III BSC resting on top of the microscope stage, ventilation and negative pressure within this BSC may affect the speed of acquisition and the stability of the images acquired overtime during an experiment. In addition, the objective holder is connected to the class III BSC by a flexible PVC membrane that could reduce the performance of the Z motor. To test the impact of the setup on the performance of our imaging system, we acquired different datasets typical of the type of experiments usually performed in MPM with the class III BSC installed or not. More precisely, we acquired a 2D mosaic (3*3 images) to test x and y axis movement; a 3D stack (14 images in the Z axis) to test z axis movement; and 3D stack time-lapse to test image stability overtime (15 cycles). The acquisition of these datasets was performed with a non-infectious sample consisting of *Convallaria majalis* rhyzome sections in order to be able to compare the imaging of the same specimen with and without the class III BSC in our BSL3 laboratory. Moreover, this type of sample is less prone to focus drift due to movement of the specimen, thus the main source of variability derives from the presence or absence of a class III BSC (Fig. 3a). As a measure of system performance, we used either the speed of acquisition to address the impact of the class III BSC on the movement of the stage in x, y and z axis, or calculated the Structural SIMilarity (SSIM) index^29^ between the maximal intensity projection of the first 3D stack and each of the subsequent ones within the same time-lapse sequence to quantify image stability overtime. Our results show that the speed of acquisition of a 2D mosaic (21,6 ± 0,74 s with isolator *vs* 22,03 ± 0,78 s (s.e.m.) without isolator) and 3D stack (14 images) (13,67 ± 0,01 s with isolator *vs* 13,62 ± 0,01 s (s.e.m.) without isolator) in our system was not affected by the presence of the BSL3 setup (Fig. 3b,c). Likewise, the stability of a 3D stack acquired overtime was largely unaffected by the BSL3 setup (SSIM index 0,949 ± 0,003 with isolator *vs* 0,931 ± 0,002 (s.e.m.) without isolator) (Fig. 3d). Hence, our BSL3 setup complies with biosafety regulations without undermining our MPM imaging systems’ performance.

In order to demonstrate one of the many applications made possible by our MPM BSL3 setup, we infected a transgenic mouse line obtained from the cross of Tg(Csfr1-eCFP)^15^ (a.k.a. Macblue) x Tg(CD11c-YFP) mice^17^ with a virulent strain of *Mycobacterium tuberculosis* (Mtb) (H37Rv, a class 3 pathogen) expressing the DsRed fluorescent protein. This model allows us to visualize different immune cells types in the lung due the expression of CFP and YFP fluorescent proteins driven by Csf1r and YFP gene promoters, respectively. In naïve mice, the majority of Macblue+ lung cells are monocytes (CD11b^high^ Gr1^low/int^), followed by alveolar macrophages (AM) (F4/80^high^ CD11c^high^) and neutrophils (CD11b^high^ Gr1^high^), while Cd11c-YFP+ lung cells are mainly AM and dendritic cells (DCs) (CD11c^high^ MHCII^high^) (Fig. 3e and Supplementary Fig. S4). By contrast, 36 days after intranasal infection by 1000 cfu of H37Rv-DsRed Mtb the majority of Macblue+ lung cells are monocytes and DCs and Cd11c-YFP+ lung cells are mainly DCs. These results indicate that significant numbers of DC precursors, which include monocytes and pre-DCs^30, 31^, have been recruited to the infected lung during infection and have locally differentiated into mature DCs. In regard of the distribution of these cells in the lung, Macblue+ and CD11c-YFP+ lung cells form several agglomerates after infection. These structures bear resemblance to granulomas, a hallmark of Tuberculosis, which are agglomerates of cells that are thought to wall off Mtb and prevent dissemination^32^. In contrast, Macblue+ and CD11c-YFP+ lung cells are relatively sparse in a naive lung (Fig. 3f). These results show that the use of this transgenic mouse line allows imaging of several lung innate immune cells known to play a key role during to Mtb infection. Indeed, DCs are essential for the control of Mtb through their ability to activate T cell immunity^33^ and infected monocytes disseminate the bacilli from the lung to other organs^21^. In order to gain a better insight into the behavior of monocytes and DCs within the Mtb infected lung, we decided to visualize them after infection by the H37Rv-Dsred virulent Mtb strain using vibratome lung tissues slices maintained in temperature regulated medium as shown before^34^. Of note, we observed that MPM at high negative pressure values (90 Pa) affected the image stability of lung slices (Supplementary Movie 1). We believe that this is due to the fact that the air flow needed for maintaining a negative pressure at 90 Pa induces agitation of the media in which the lung slice is immersed and the specimen itself. Indeed, *Convallaria majalis* rhyzome sections imaged at the same pressure levels did not show issues with image stability (Fig. 3a–d). In line with this assumption, MPM of lung slices at 20 Pa is not affected by image stability issues and has been chosen as a negative pressure value to perform MPM imaging. Moreover, the cell motility parameters were similar to those obtained by others^35–37^, despite the lack of oxygen regulated media. Our results show that Mtb infection had a differential impact on the migratory behaviors of Macblue+ and CD11c-YFP+ lung cells. Indeed, Macblue+ lung cells migrated with increased average speed in an Mtb-infected lung compared to in a naive lung (0,92 μ/min vs 0,38 μ/min, p < 0,0001) whereas the increase in average migration speed of CD11c-YFP+ was less pronounced (0,87 μ/min vs 0,68 μ/min, p = 0,01) (Fig. 3g, Supplementary Movies 2–4). By contrast, the migration of Macblue+ in an H37Rv-infected lung was more sinuous as shown by their decreased meandering index (the ratio of the displacement of a cell to the total length of the path that the cell has travelled) in comparison to a naive lung (0,20 vs 0,25, p = 0,01). Even though, this effect is more pronounced in CD11c-YFP+ cells (0,20 vs 0,38, p < 0,0001). An increase in migration speed of Macblue+ cells, despite a more tortuous path of migration, allowed them to cover larger territories during Mtb infection than in a naïve lung. Meanwhile CD11c-YFP+ cell migration was also more tortuous after Mtb infection and only accompanied by small increase in migration speed, which lead to smaller territories covered during their migration (Fig. 3i). Altogether, these results show Mtb infection preferentially promotes the motility of Macblue+. The fact that Mtb infection preferentially promotes the migration speed on Macblue+ cells’ (49% are monocytes) but not on CD11c-YFP+ cells (63% are DCs) may favor the capture of Mtb by monocytes and entry into lymphatics vessels that that are exit routes within the lung to reach the draining lymph nodes^38, 39^. Hence providing a possible explanation for the fact that monocytes are essential for the dissemination of the Mtb bacilli from the lung to other organs but not DCs^21^.

## Discussion

Our BLS3 biocontainment strategy for MPM allows *in vivo* studies with highly virulent human pathogens without compromising neither the performances of the imaging setup nor its regular maintenance by the manufacturers. Importantly, the basic principles of our BSL3 setup can be easily applied to other MPM or other fluorescence microscopy systems from different manufacturers with minor adjustments, but not to transmitted light microscopy. For MPM or other fluorescence microscopes the shape and size of BSCs need to be adapted to the microscope stage and type of laser. For transmitted light microscopy other solutions need to be developed. Altogether, we trust that our approach will promote the development of scientific programs involving fluorescence microcopy imaging approaches of highly virulent human pathogens that will lead to a better understanding of the reasons for the success of these microorganisms in causing disease in the human population.

## Material and Methods

### Multiphoton Microscope

The laser laser scanning microscope system is a commercially available integrated setup (TRiMScope, LaVision BioTec GmbH, Bielefeld, Germany). An automatic tunable Ti:Sa laser (Chameleon Ultra II, Coherent) generating pulsed radiation in the range of 680–1080 nm is used as excitation light source for conventional MPM. The beam control (BeamOptimizer, Lavision BioTec) uses a polarizer/analyzer as attenuation and an electro optical Modulator for fast beam switching for line blanking and phototreatment applications. A telescope adapts the beam diameter for the scanhead. Positon sensitive 4Q-diodes help alignment control of the laser beam into the scanhead. Inside the scanhead, light passes a variable three-lens telescope that is used to adapt the beam diameter to the objective lens entrance aperture. Scanning is done with a conventional pair of galvanometer scanners that allow a free selection of the position and size of the field of view. An objective lens from Olympus, 25x, NA 1.05 water immersion features a field of view up to 400 × 400 µm @2.5 Hz frame rate, 800 Hz line frequency and 250 × 250 pix resolution down to 40 × 40 µm @ 2600 Hz line frequency. The scanhead works on two microscope stands: For upright imaging the system is equipped with a Zeiss Axio Examiner illuminator, the inverted imaging can be done on a Nikon Ti-U commercial system. An optical switch can use the microscope stands alternately. Both microscope stands are equipped with motorized stages. The sample height position can be adapted in a wide range of 80 mm travel range manually based on hydraulic pistons. XY movement is motorized (50 mm range) for automatic image stitching application (IVS stage, LaVision BioTec GmbH). The inverted microscope system is equipped with a shifting table V540 (Luigs & Neumann GmbH, Ratingen, Germany), which can be also adapted in height by a hydraulic system (LaVision BioTec) about 80 mm. Multiphoton fluorescence is collected by the objective lens and can either be viewed directly through the eyepieces or imaged onto the detection part of the system. In backward direction the emitted photons are collected and detected by non-descanned photomultipliers. A multi PMT detector with switchable dichroic mirrors allows the spectral separation of the fluorescence up to six channels. In our experiment each microscope system is equipped with two modified GaAsP high sensitivity photomultiplier modules (LaVision BioTec, Hamamatsu H 7422-40) and 2 standard photomultiplier modules (Hamamatsu H 6780-20). Controlling imaging parameters and steering of all devices is integrated into one software package (Imspector, LaVision BioTec GmbH).

### Class III BSC

PVC-U sheets from Richardson, France were used to construct the BSC that was equipped with a glove box system from Piercan, France and Eurobioconcept, France. A 160 mm ventilator from Ebmpapst, France (G2E160-AY47-01) ensured the ventilation of this class III BSC with room air that was filtered by a HEPA filter (Camfil, France). The ventilated air was pulsed into the BSC through 63 mm flexible tubing from Flexadux, France and heated by a heating unit from Acim Jouanin, France.

### Class I BSC

PVC-U sheets from Richardson, France were used to construct the BSC from the laser head and power supply. A 120 mm ventilator from Ebmpapst, France (G2E120-AR77-01) ensured the ventilation of both laser head and power supply class I BSC with room air that was filtered by a HEPA filter (Camfil, France). Ventilated air was directed to the BSC through 63 mm flexible tubing from Flexadux, France and connected to the BSC by a manual valve from FIP, France. A pressure probe was inserted into the laser head class I BSC to monitor pressure levels. A glove box system from Piercan, France and Eurobioconcept, France was added to the power supply class I BSC in order to allow the operation of the power supply controls. Power, USB, Serial port and cooling liquid cables were pass through the power supply BSC by an individual thermoretractable cable glands from Farnell, France. A set of wheels was added to the power supply BSC to the movement in and out of the facility.

### Class III Biosafety Cabinet decontamination procedure

H_2_O_2_ was pulsed into both upright and inverted Class III BSC by a Clarus L2 decontamination system from Bioquell at a rate of 3 g/minute for 2 hours that resulted in a peak of concentration of 450 ppm during incubation. Prior to decontamination, strips containing 2.2 × 10^6^ colony forming units (cfu) of *Geobacillus stearothermophilus* spores (ATCC #12980) were positioned at 5 different positions within this class III BSC. At the end of the decontamination cycle these strips were incubated for 7 days in Tryptic Soy Broth at 55 °C to evaluate the viability of *G*. *stearothermophilus* spores, thus the efficiency of the decontamination procedure.

### Room requirement for BSL3 MPM setup

The microscope is mounted on a 3100 × 1200 mm Newport optical table and is house in a 17 m^2^ room with a celling height of 2.6 m. The entire is kept at a pressure of −75 Pa and a temperature between 20–24 °C.

### Mouse infection

Six- to eight-week-old female Macblue x CD11c-YFP mice were anesthetized with a cocktail of ketamine (60 mg/kg; Merial) and xylasine (10 mg/kg; Bayer) and infected intranasally with 1000 CFUs of a virulent *Mycobacterium tuberculosis* strain (H37Rv) expressing DsRed fluorescent protein in 25 μl of PBS-0.01% Tween 80. Prior to infection, *M*. *tuberculosis* H37Rv-DsRed was grown from a frozen stock to mid-log phase in 7H9 medium (BD) supplemented with albumin-dextrose-catalase (ADC, Difco) in the presence of 50 μg/ml of kanamycin. Animal procedures were conducted in strict accordance with French laws and regulations in compliance with the European community council directive 68/609/EEC guidelines and its implementation in France. All animal procedures were reviewed and approved by an Ethical Committee for Animal Experimentation and obtained an authorization from the Ministere de l’Éducation Nationale, de l’Enseignement Supérieur et de la Recherche (reference 2015031015173748, Apafis#295).

### Flow cytometry

Mice were euthanized by Pentobarbital i.p. injection (200 mg/kg) the lungs harvested and placed in PBS. Tissues were minced using GentleMacs Dissociator, then incubated in 1 mg/ml collagenase D (Roche) and 30 μg/ml DNase I (Roche) at 37 °C for 30 min. Each tissue was forced through a 40-μm cell strainer (BD Falcon) prior to antibody staining. Staining for surface markers was done by suspending an aliquot of lung cells in medium with an Fc receptor-blocking Ab (clone 2.4G2) for 10 min at RT followed by washing and resuspension with CSB buffer (PBS, 1% FCS, 0.1% sodium azide, and 1 mM EDTA) containing Abs and incubation at RT for 30 min. Cells were then washed twice and fixed in 4% paraformaldehyde for 2 h at 4 °C. Samples were acquired using a BD LSRII. Antibodies were purchased from Ozyme, France otherwise stated. Antibodies conjugates were CD11b-PECy7, Gr1 APC-Cy7, MHCII Alexa-700, F4/80 Brilliant Violet 605, CD11c Alexa-647.

### MPM lung imaging

Mice were euthanized by Pentobarbital i.p. injection (200 mg/kg) and lungs processed as described previously by Thronton, EE *et al*.^37^. Briefly, lungs were inflated with 2% low melting temp agarose in sterile PBS, excised and put at 4 °C until agarose has solidified. Next, the left lobe was isolated, cut into ∼900-µm sections using a Leica VT1000S vibratome filled with cool PBS and mounted on plastic petri dish with Vetbond (3 M) containing RT RPMI without phenol red that was thermo-regulated at 37 °C during imaging sessions but not oxygenated. Samples were excited with a Coherent Ultra II Ti:Sapphire laser tuned to a wavelength of 900 nm, and emission wavelengths of 450/50 nm (for CFP), 525/50 nm (for YFP), and 620/60 nm (for DsRed) were collected. For time-lapse acquisition, x, y stacks spaced 1 µm apart in z axis were acquired over several minutes with a 25x NA 1.05 Objective (Olympus).

### Image Analysis

Images were analyzed with Imaris software (Bitplane) using a Matlab algorithm to separate CFP and YFP signals and the spot tracker application to obtain 3D velocity and meandering index parameters for each track. MSD, Track and Turning angle plots were obtained using a previously described custom script in Matlab (MathWorks)^40^.

### Statistic analysis

Significance of differences were calculated using an unpaired Student’s T test with the help of GraphPad Prism software.

## Electronic supplementary material

Supplementary Figures and Movie Captures

Macblue and CD11c-YFP cell migration in lung after M. tuberculosis infection at high negative pressure levels (90Pa).

Macblue and CD11c-YFP cell migration in the lung before infection.

Macblue and CD11c-YFP cell migration in lung after M. tuberculosis infection at low negative pressure levels (20Pa).

Macblue and CD11c-YFP cell migration in lung after M. tuberculosis infection at low negative pressure levels (20Pa) and high zoom.
