# Supplementary material for: Biosafety Level 3 setup for multiphoton microscopy in vivo
Source: Sci Rep. 2017 Apr 3;7:571. doi: 10.1038/s41598-017-00702-x (PMC5429670; doi:10.1038/s41598-017-00702-x)
Supplement: Supplementary file 1 — Supplementary Figures and Movie Captures [file 41598_2017_702_MOESM1_ESM.pdf]

Supplemental Information

Biosafety Level 3 setup for multiphoton microscopy *in vivo*

Barlerin D[3], Bessiere G[3], Domingues J[1,2], Schuette M[4], Feuillet C[4], Peixoto A[1,2,*]

[1] Centre National de la Recherche Scientifique (CNRS), Institut de Pharmacologie et de Biologie Structurale (IPBS), 31000 Toulouse, France

[2] Université de Toulouse, Université Paul Sabatier, Institut de Pharmacologie et de Biologie Structurale (IPBS), 31000 Toulouse, France

[3] EuroBioconcept SAS, 94380 Bonneuil sur Marne, France

[4] Lavision Biotech GmbH, 33617 Bielefeld, Germany

[*] corresponding author:

Institut de Pharmacologie et de Biologie Structurale (IPBS)

31000 Toulouse, France

+33 561175833

antonio.peixoto@ipbs.fr

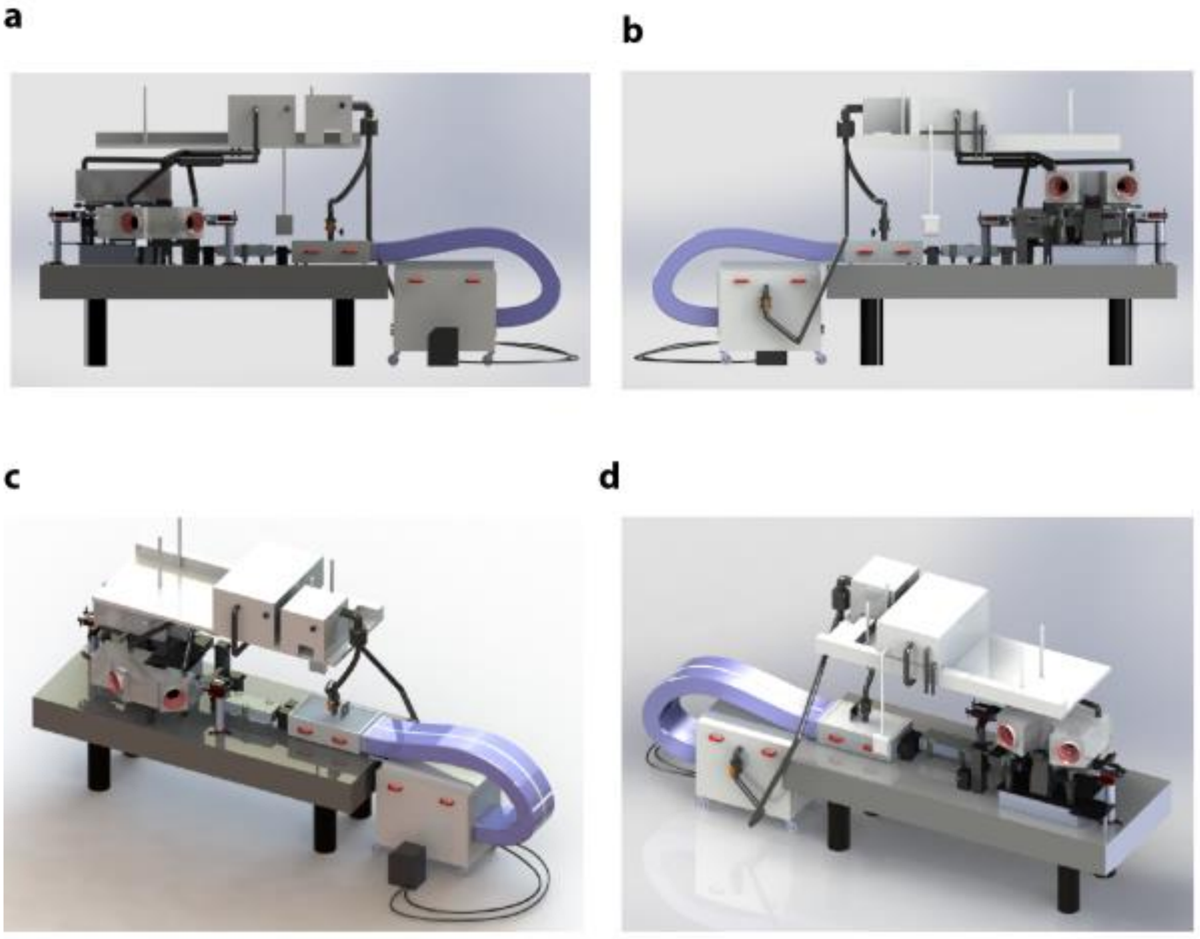

Supplementary Figure S1. 3D drawings of BSL3 setup for MPM imaging. The BSL3 setup was rendered digitally using Solidworks software for a detailed description of the assembly of all its parts. Left (a) and right (b) side view. Left (c) and right (d) orthogonal view.

Enable 3D View

Supplementary Figure S2. Interactive 3D PDF of BSL3 setup for MPM imaging. SolidWorks drawings of the BSL3 setup were converted to 3D PDF file for browsing and detailed description of the assembly of all its parts. See 3D PDF file. In order to obtain the dimensions of biosafety cabinets and other parts please use 3D measurement tool available in Adobe Reader version 11.0.1 or higher while using a scale ratio of 1 model unit = 1000mm. For more detailed instructions please read: https://helpx.adobe.com/acrobat/using/measuring-3d-objects-pdfs.html#measuring_3d_objects_in_pdfs

Supplementary Figure S3. Interactive 3D PDF of objective holder for BSL3 setup for MPM imaging. SolidWorks drawings of the BSL3 setup were converted to 3D PDF file for browsing and detailed description of the assembly of all its parts. See 3D PDF file. In order to obtain the dimensions of biosafety cabinets and other parts please use 3D measurement tool available in Adobe Reader version 11.0.1 or higher while using a scale ratio of 1 model unit = 1000mm. For more detailed instructions please read: https://helpx.adobe.com/acrobat/using/measuring-3d-objects-pdfs.html#measuring_3d_objects_in_pdfs

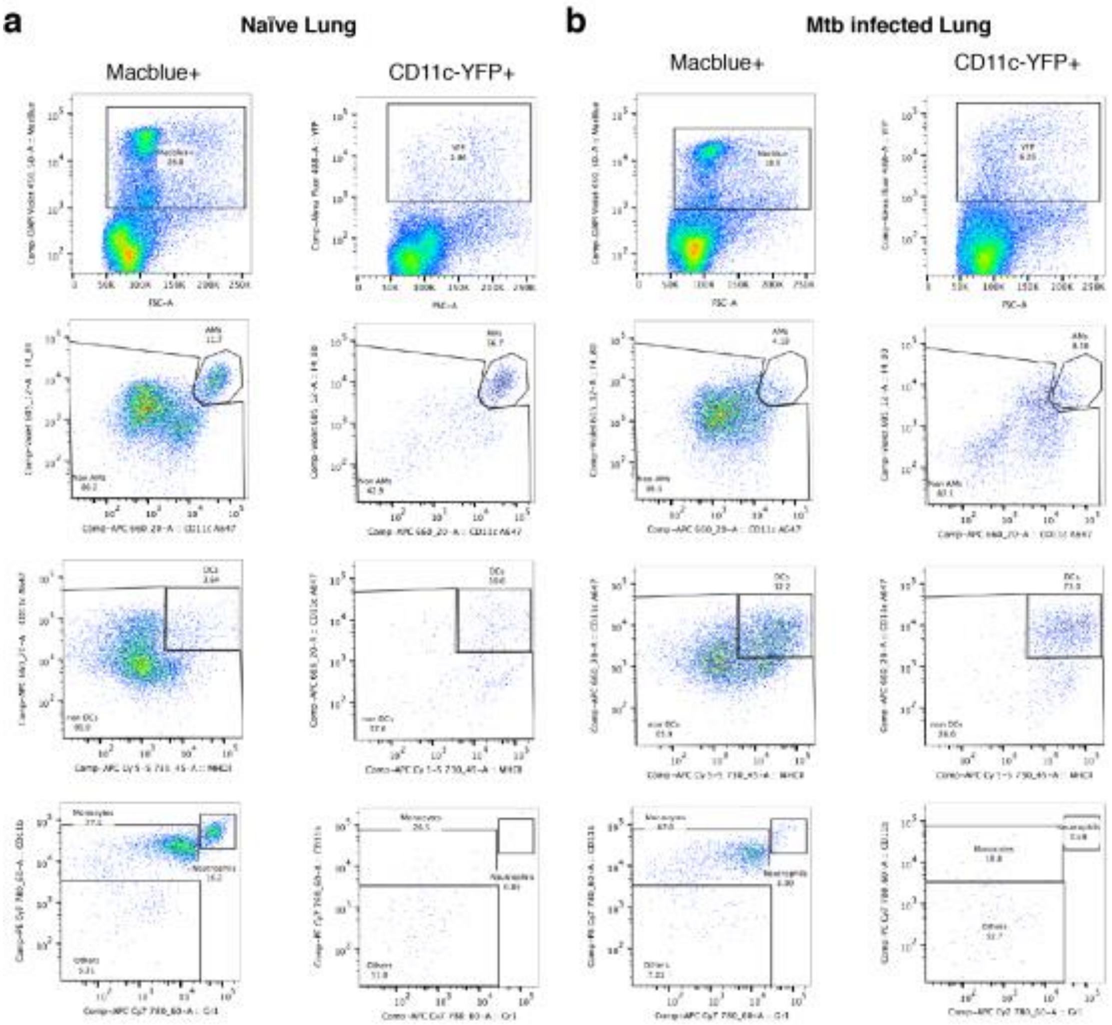

Supplementary Figure S4. Flow Cytometry gating strategy to define different immune cell types in the mouse lung. (a) naïve and (b) *Mycobacterium tuberculosis* (Mtb) infected lung. Alveolar macrophages (AM) are F4/80^{high} CD11c^{high}, Dendritic cells (DCs) are CD11c^{high} MHCII^{high}, Monocytes are CD11b^{high} Gr1$^{low/int}$ and Neutrophils are CD11b^{high} Gr1^{high}.

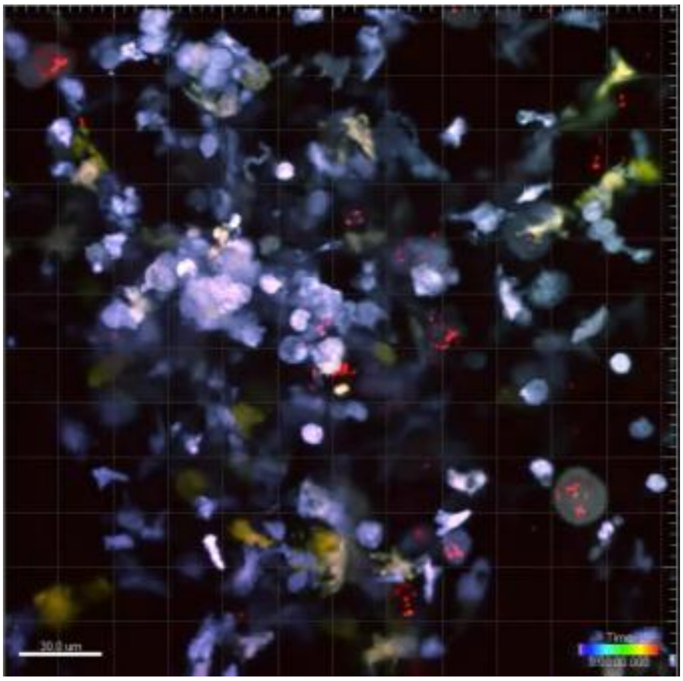

Supplementary Movie 1. **Macblue and CD11c-YFP cell migration in lung after *M. tuberculosis* infection at high negative pressure levels (90Pa)**. Macblue x Cd11c-YFP mice were infected with H37Rv-Dsred and 36 later lung slices were obtained using a vibratome and used for MPM. 107μm 3D stack. See Sup Movie 1 movie file.

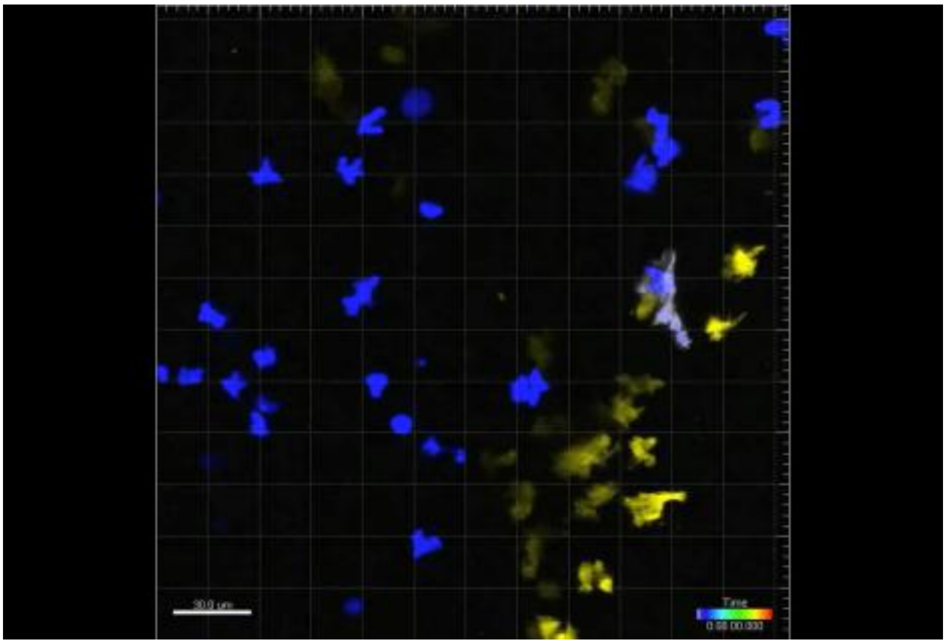

Supplementary Movie 2. Macblue and CD11c-YFP cell migration in the lung before infection. Macblue x CD11c-YFP mice were sacrificed and lung slices obtained using a vibratome for MPM. 44μm 3D stack. See See Sup Movie 2 movie file.

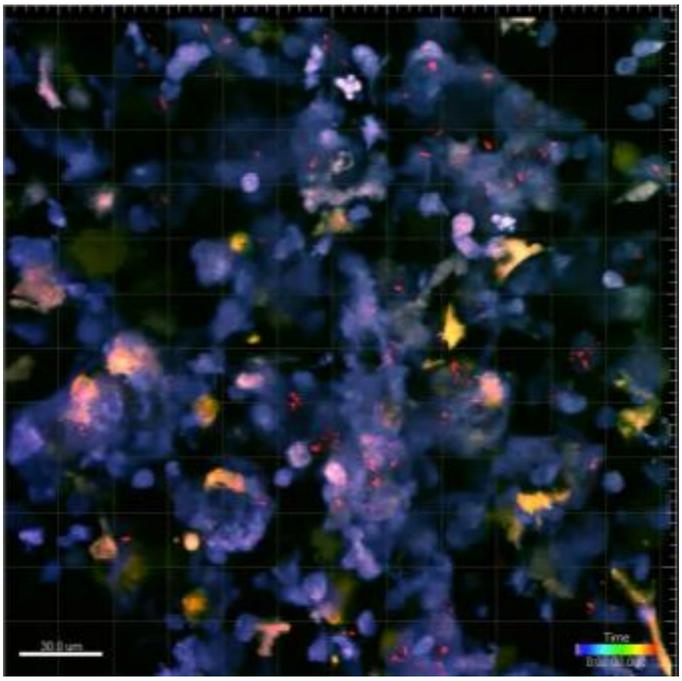

Supplementary Movie 3. Macblue and CD11c-YFP cell migration in lung after *M. tubercul*osis infection at low negative pressure levels (20Pa). Macblue x Cd11c-YFP mice were infected with H37Rv-Dsred and 36 later lung slices were obtained using a vibratome and used for MPM. 90µm 3D stack. See Sup Movie 3 file.

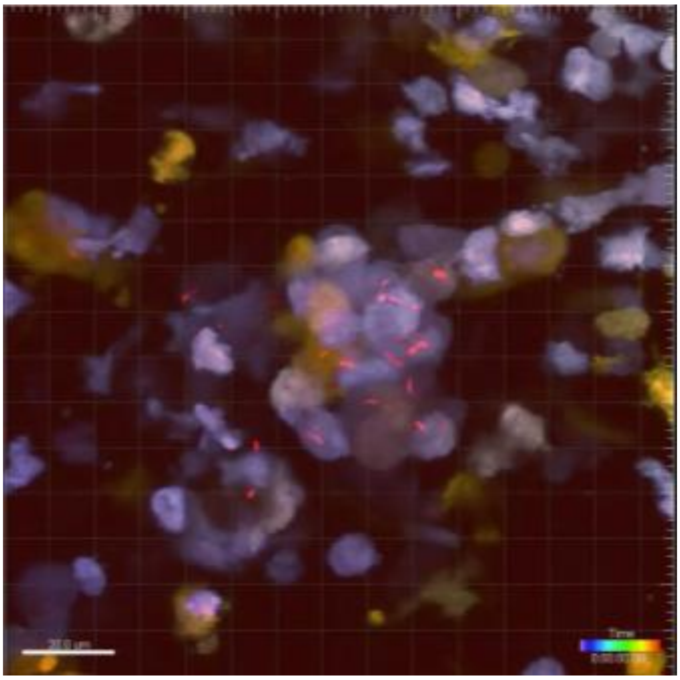

Supplementary Movie 4. Macblue and CD11c-YFP cell migration in lung after *M. tubercul*osis infection at low negative pressure levels (20Pa) and high zoom. Macblue x Cd11c-YFP mice were infected with H37Rv-Dsred and 36 later lung slices were obtained using a vibratome and used for MPM. 67μm 3D stack. See Sup Movie 4 file.
